# Effect of *N*-acetylcysteine on serum thyroid hormone levels in nonthyroidal illness syndrome

**DOI:** 10.1186/cc12653

**Published:** 2013-06-19

**Authors:** J Vidart, SM Wajner, BD Schaan, AL Maia

**Affiliations:** 1 Hospital de Clínicas de Porto Alegre, Santa Cecília, Porto Alegre, RS, Brazil

## Introduction

Nonthyroidal illness syndrome (NTIS) refers to changes in thyroid hormone levels affecting up to 75% of critically ill patients. Cytokines and oxidative stress have been implicated as causative factors, as they derange deiodinase reactions. Interestingly, the addition of *N*-acetylcysteine (NAC) in a cell model prevented the effect of IL-6 on deiodinases, probably through a mechanism that restores catalytic activity of the enzyme [1]. NTIS is an independent marker of poor prognosis during myocardial infarction (MI) [2]. Here, we investigate whether NAC administration would prevent the decrease in serum thyroid hormone levels observed in patients with MI.

## Methods

This was a randomized, multicenter clinical trial. Patients with MI within 12 hours of evolution who underwent primary percutaneous coronary intervention were eligible. Patients were randomized to receive NAC (1,200 mg, intravenous, every 12 hours) or placebo for 48 hours. Baseline characteristics, clinical history and serial blood samples for measurements of thyroid hormones were collected. Primary outcome was the variation of serum T3 levels. Statistical tests used included χ^2 ^test analyses for proportions and Student's *t *test analyses for means.

## Results

**Figure 1:**
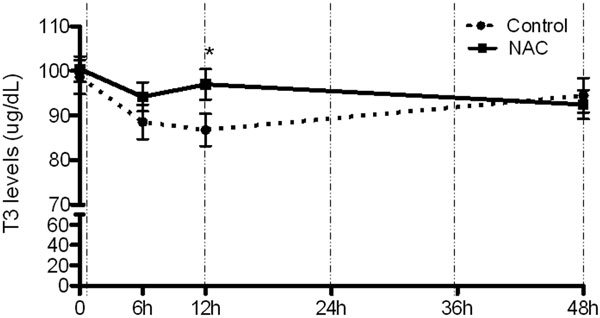


Sixty-seven patients were included. There were no differences between groups with respect to baseline clinical characteristics. When compared with baseline, the levels of serum T3 decreased in the placebo group at 12 hours of follow-up (98.6 vs. 86.8 μg/dl, *P *= 0.001), as expected for the NTIS. Interestingly, the T3 descendent curve was attenuated in patients who were randomized to NAC (100.4 vs. 96.9 μg/ dl, *P *= 0.396) (Figure 1). Similar serum T3 levels were observed in both groups at 48 hours and on the 5th day. Serum TSH levels were virtually identical between the two groups. In the comparison between groups, the mean T3 serum levels were higher in the NAC treatment group at 12 hours than in the placebo group (*P *= 0.045).

## Conclusion

This is the first clinical trial designed to investigate the effect of NAC on NTIS in humans. We show that patients with myocardium infarction who received NAC treatment on admission had attenuation of the degree of T3 level decrease compared with those who received placebo. As expected, NAC did not interfere with central feedback, as TSH levels presented a similar rise in both groups, probably as a marker of recovery from acute illness. However, whether this effect is able to prevent mortality or to ameliorate patient outcome remains to be elucidated.
